# Field‐Induced Antiferromagnetic Correlations in a Nanopatterned Van der Waals Ferromagnet: A Potential Artificial Spin Ice

**DOI:** 10.1002/advs.202409240

**Published:** 2024-12-08

**Authors:** Avia Noah, Nofar Fridman, Yishay Zur, Maya Markman, Yotam Katz King, Maya Klang, Ricardo Rama‐Eiroa, Harshvardhan Solanki, Michael L. Reichenberg Ashby, Tamar Levin, Edwin Herrera, Martin E. Huber, Snir Gazit, Elton J. G. Santos, Hermann Suderow, Hadar Steinberg, Oded Millo, Yonathan Anahory

**Affiliations:** ^1^ The Racah Institute of Physics The Hebrew University Jerusalem 9190401 Israel; ^2^ Center for Nanoscience and Nanotechnology The Hebrew University Jerusalem 91904 Israel; ^3^ Faculty of Engineering Ruppin Academic Center Emek‐Hefer Monash 40250 Israel; ^4^ Institute for Condensed Matter Physics and Complex Systems School of Physics and Astronomy University of Edinburgh Edinburgh EH93FD UK; ^5^ Imperial College London, Blackett Laboratory London SW7 2AZ UK; ^6^ Laboratorio de Bajas Temperaturas Unidad Asociada UAM/CSIC Departamento de Física de la Materia Condensada Instituto Nicolás Cabrera and Condensed Matter Physics Center (IFIMAC) Universidad Autónoma de Madrid Madrid E‐28049 Spain; ^7^ Departments of Physics and Electrical Engineering University of Colorado Denver Denver CO 80217 USA; ^8^ The Fritz Haber Research Center for Molecular Dynamics The Hebrew University of Jerusalem Jerusalem 91904 Israel; ^9^ Donostia International Physics Center (DIPC) Donostia‐San Sebastián Basque Country 20018 Spain; ^10^ Higgs Centre for Theoretical Physics University of Edinburgh Edinburgh EH93FD UK

**Keywords:** artificial spin ice, atomistic spin dynamics, CrGeTe_3_, magnetic interactions, nano magnetism, scanning SQUID microscopy, vdW magnets

## Abstract

Nano‐patterned magnetic materials have opened new venues for the investigation of strongly correlated phenomena including artificial spin‐ice systems, geometric frustration, and magnetic monopoles, for technologically important applications such as reconfigurable ferromagnetism. With the advent of atomically thin 2D van der Waals (vdW) magnets, a pertinent question is whether such compounds could make their way into this realm where interactions can be tailored so that unconventional states of matter can be assessed. Here, it is shown that square islands of CrGeTe_3_ vdW ferromagnets distributed in a grid manifest antiferromagnetic correlations, essential to enable frustration resulting in an artificial spin‐ice. By using a combination of SQUID‐on‐tip microscopy, focused ion beam lithography, and atomistic spin dynamic simulations, it is shown that a square array of CGT island as small as 150 × 150 × 60 nm^3^ have tunable dipole–dipole interactions, which can be precisely controlled by their lateral spacing. There is a crossover between non‐interacting islands and significant inter‐island anticorrelation depending on how they are spatially distributed allowing the creation of complex magnetic patterns not observable at the isolated flakes. These findings suggest that the cross‐talk between the nano‐patterned magnets can be explored in the generation of even more complex spin configurations where exotic interactions may be manipulated in an unprecedented way.

## Graphical abstract

CrGeTe₃ nanoislands exhibiting tunable dipolar interactions resulting in an effective inter‐island antiferromagnetic coupling are demonstrated. The crossover between random and anticorrelated islands depends on their spatial distribution. The dipolar interaction enables the formation of complex magnetic patterns, which can be harnessed to generate more intricate spin configurations including artificial spin ice in van der Waals materials.

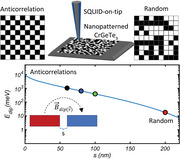

## 1 Introduction

Magnetic order is the result of an interplay between magnetic exchange, magnetic anisotropy, Zeeman coupling to an external field, and the dipolar interaction. Although the last is typically the weakest term, the dipolar interaction can become significantly large under certain conditions. For instance, the exchange interaction vanishes when magnetic particles are separated. In addition, the anisotropy energy can be reduced by applying an external magnetic field, which suppresses the anisotropy barrier near the coercive field. In this scenario, the dipolar interaction becomes substantial. Such conditions are theoretically met in single‐molecule magnets during the magnetization reversal process, where dipolar interaction is expected to cause magnetic self‐ordering.^[^ ^1^, ^2^, ^3^ ^]^ The absence of experimental evidence may be partially due to the small spatial scales and the incompatibility of most surface magnetic microscopy techniques with the surface quality of crystallized single‐molecule magnets. To circumvent this problem, extensive research has been conducted on larger magnetic particles lithographically patterned from thin films.^[^ ^4^, ^5^, ^6^ ^]^ That platform was used to study a broad variety of magnetic lattices such as 1D chains,^[^ ^7^ ^]^ 2D frustrated arrays,^[^ ^8^, ^9^, ^10^ ^]^ and 3D lattices.^[^ ^11^, ^12^ ^]^ However, until now this platform was limited to rather simple magnetic thin films such as cobalt or permalloy.

Recent advancements in 2D materials have unveiled the potential of magnetically ordered van der Waals (vdW) compounds,^[^ ^13^, ^14^, ^15^, ^16^ ^]^ with particular interest in the effect of spatial confinement on the magnetic order.^[^ ^17^, ^18^ ^]^ There have also been recent reports that confinement causes a transition from soft to hard ferromagnetism in Fe_3_GeTe_2_,^[^ ^19^ ^]^ CrSiTe_3_,^[^ ^20^ ^]^ and CrGeTe_3_ (CGT).^[^ ^21^ ^]^ In addition, ferromagnetism has been observed in atomically thin layers.^[^ ^22^ ^]^ These results are surprising given that magnetism is expected to be weaker in confined structures where, as pointed out by Mermin and Wagner, thermal fluctuations might be expected to suppress long‐range ordering in the absence of anisotropy.^[^ ^23^ ^]^ However, such an assumption is only valid in the thermodynamic limit where samples beyond the size of the known universe^[^ ^24^ ^]^ would hold such dependence of the anisotropy to generate magnetism in 2D. Specifically, it has been experimentally demonstrated that thin CGT films (*d* < 10 nm) exhibit a net magnetization at zero applied field.^[^ ^21^, ^25^ ^]^ In contrast, the interior of thicker flakes (*d* > 10 nm) has zero net magnetization, with hard ferromagnetism appearing only at the sample edges.^[^ ^21^, ^26^ ^]^ These findings raise the question of the existence of edge effects in the 0D limit, as well as the option that finite‐size magnets may be manipulated in storage technology applications. It is currently unknown whether nano‐flakes of 2D magnets disposed in patterned recording media, i.e., magnetic islands distributed in a grid, could provide any cooperative phenomena useful for manipulating spins in an information bit.

Here, we employ Ga^+^ FIB amorphization of CGT flakes to fabricate an array of square‐shaped magnetic islands with an effective inter‐island antiferromagnetic (AFM) interaction which is the essence of artificial spin‐ice arrays. Scanning SQUID‐on‐tip (SOT) microscopy^[^ ^26^, ^27^ ^]^ is then employed to investigate the magnetic properties of the arrays as a function of the applied field. Our results indicate that the magnetic islands remain fully magnetized at zero applied magnetic field down to the smallest achievable size of ≈ 150 × 150 × 60 nm^3^, which is surprising considering that a pristine isolated CGT flake of 60 nm thickness does not hold a net magnetization in these conditions.^[^ ^21^ ^]^ The use of an external field to suppress the anisotropy barrier and reach the coercive field of the magnetic array revealed that the dipolar field causes significant AFM correlations to emerge. Atomistic spin dynamics reproduced closely the effect also indicating that zero‐field approaches where the islands are distributed much closer displayed similar anticorrelation features strongly mediated by the stray fields across the magnetic grid. Our results pave the way to study magnetism in nanopatterned arrays of 2D magnets opening a pathway to explore a large number of complex configurations, interactions so far not accessible in vdW materials such as artificial spin‐ice systems, and their vast implications.

## 2 Results

**Figure 1:**
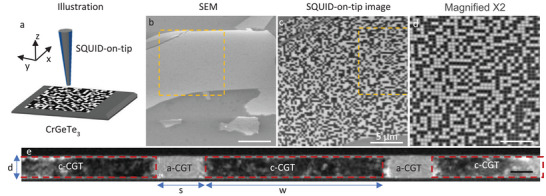
Stable CrGeTe_3_ magnetic nanoisland array. a) Schematic illustration of the experimental setup depicting the SQUID‐on‐tip (SOT) and the sample. b) Scanning electron microscope image of CrGeTe_3_ (CGT) patterned using a Ga^+^ focused ion beam into a grid with an island size of 230 × 230 × 30 nm^3^. The area marked with an orange square is shown in c. The scale bar is 10 µm. c,d) *B_z_* (*x*,*y*) SOT images acquired at µ_0_ *H_z_* = 85 mT. The area marked with an orange square in (c) is shown in (d). The SOT images are 20 × 20 and 10 × 10 µm^2^ c,d) with a scale bar corresponding to 5, and 2.5 µm c,d). All images were acquired with 35 nm pixel size and acquisition time of 20 min/image. The black/white color scale represents magnetic moments pointing down/up. e) Cross‐section scanning transmission electron microscopy of the CrGeTe_3_ (CGT) etched array. The gray area is amorphous (a‐CGT), and the dark area is crystalline (c‐CGT). The scale bar is 30 nm.

CGT flakes with thicknesses ranging from 30 to 110 nm were exfoliated on top of a SiO_2_‐coated Si wafer. **Figure** **1**b presents a scanning electron microscope (SEM) image of a CGT flake. Using a 30 keV Ga^+^ FIB, we amorphized vertical and horizontal lines to fabricate an array of square‐shaped magnetic islands (Figure 1a). Figure 1e shows a cross‐sectional scanning transmission electron microscopy (STEM) image confirming that certain portions of the CGT flake were amorphized (a‐CGT, Figure 1e). As demonstrated in previous work,^[^ ^28^ ^]^ amorphous CGT is nonmagnetic, thereby impeding exchange coupling between the islands. Each magnetic island in the presented array has dimensions of *w* × *w* × *d*  =  230 × 230 × 30 nm^3^ and a separation of *s*  =  70 nm. We resolve the local out‐of‐plane component of the magnetic field, *B_z_* (*x*,*y*), of single‐islands at 4.2 K (Figure 1a) by using a scanning SOT microscope (see Experimental Section). Figure 1c,d present SOT images of *B_z_* (*x*,*y*) of a magnetic array acquired while applying an external magnetic field of μ_0_ *H_z_* = 85 mT, where a black color‐coded area indicates islands with a magnetic moment pointing down, while a white color‐coded area indicates islands with moments pointing up. The images, which have dimensions of 20 × 20 µm^2^ (Figure 1c) and 10 × 10 µm^2^ (Figure 1d) include over 5 000 and 1 000 islands, respectively. The stable magnetic signal demonstrates the magnetic island stability at the nanoscale.

**Figure 2:**
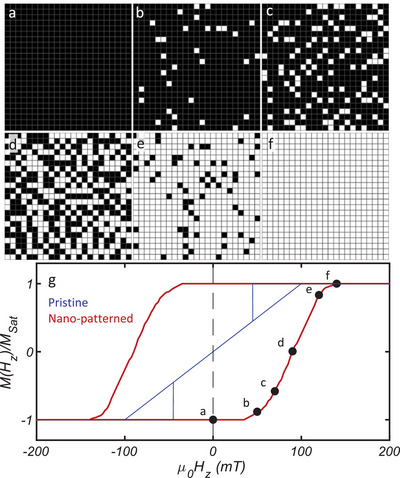
Magnetic field response of the CrGeTe_3_ array magnetization. a–f) The sequence of binary matrices computed from the SQUID‐on‐tip (SOT) *B_z_* (*x*,*y*) images at distinct values of an applied out‐of‐plane field µ_0_ *H_z_*. The SOT image sizes are 8 × 8 µm^2^. The black/white color scale represents the magnetic moments pointing antiparallel/parallel (down/up) to the applied field. The images were acquired at µ_0_ *H_z_* = 0, 50, 70, 90, 120, and 150 mT a,b,c,d,e,f). g) Magnetization curves *M*(*H_z_*) of nano‐patterned (red) and pristine (blue) 30 nm thick CrGeTe_3_ flake, in relation to the saturation magnetization, *M_Sat_*, extracted from the SOT images.

The response of the patterned CGT array to an applied out‐of‐plane magnetic field, *H_z_*, is shown in **Figure** **2**a–f, which presents a set of 8 × 8 µm^2^ SOT images comprising 529 islands that have been converted into binary images for clarity. The images were acquired at fields ranging between 0 ≤ µ_0_ *H_z_* ≤  *H_s_* =  150 mT, following a negative field excursion at µ_0_ *H_z_* =   − 200 mT. The array field evolution is shown in Movies S1,S2 (Supporting Information). The SOT image shown in Figure 2a demonstrates that at µ_0_ *H_z_* =  0, each island remains magnetized in the direction of the previously applied field (negative). As *H_z_* increases, the number of islands with magnetization parallel to the field grows at the expense of the number of islands pointing antiparallel to it, until the saturation field, *H_s_*, is reached and all the islands point in the positive direction. To quantify the magnetization (*M*(*H_z_*)) of the array, we measured the number of islands oriented in a specific direction. Figure 2g (red curve) depicts the magnetization curve *M*(*H_z_*) resulting from the image analysis divided by the magnetic moment of the entire array, *M_Sat_* =  *n* *m_i_*, where *n* is the number of islands and *m_i_* the magnetic moment of a single island (here *n*  =  529 and *m_i_* ≈ 5.7 × 10^6^ µ_B_).

The patterned flake possesses the typical hysteresis curve of a hard ferromagnet (*M* ( *H_z_* =  0) = *M_Sat_* ). In contrast, a pristine flake of the same thickness (*d*  =  30 nm), exhibits a zero net magnetization at *H_z_* =  0, with a bowtie‐shaped magnetization curve as measured in previous work^[^ ^21^ ^]^ (Figure 2g, blue curve). In that work, hard ferromagnetism was observed at the sample edge. Here, a comparison of the hysteretic loops reveals that confinement has the effect of hardening the ferromagnetism in nanoislands. This comparison highlights the similarity between the magnetic properties of an edge and a nanoisland, which is mainly constituted of edges. The mechanism explaining this unusual edge property remains unknown and will require further investigation. In what follows, we focus on the dipolar interactions between the islands.

**Figure 3:**
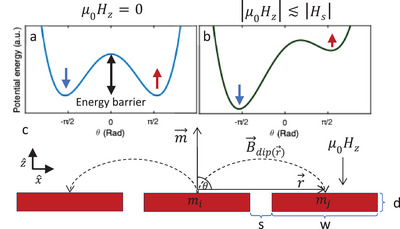
Energy scales and the dipolar energy *E_dip_*. a) Schematic of the double‐well energy potential for a ferromagnet at µ_0_ *H_z_* = *H_c_*, representing two stable states corresponding to the magnetization pointing up and down. b) Schematic of the energy potential under an external magnetic field|*H_c_* | < |µ_0_ *H_z_* | < |*H_s_* |. The blue and red arrows represent islands with magnetization pointing down and up, respectively. c) Illustration of the dipolar field ${\vec{B}}_{dip(\vec{r})}$ exerted by island *m_i_* on its neighboring island *m_j_*. The magnetic moment $\vec{m}$ points upward, and the distance vector $\vec{r}$ lies in the plane. An additional external magnetic field µ_0_ *H_z_* is applied downward.

We consider a point dipole in the island generating a dipolar field at the point $\vec{r}$ as illustrated in **Figure** **3**c. The stray field is given by the following expression ${\vec{B}}_{dip}\;\left(\vec{r}\right)=\frac{\mu_{0}}{4\pi }\;\left[\frac{3\vec{r}\left(\vec{m}\cdot \vec{r}\right)}{r^{5}}-\vec{\frac{m}{r^{3}}}\right]$, where $\vec{r}=\left(0,0,0\right)$ is the position vector of the considered dipole. In the case of a moment pointing out of the plane and neighbors situated in the plane, we obtain $\vec{r}⊥\vec{m}$, causing the first term of ${\vec{B}}_{dip}$ to vanish and thus ${\vec{B}}_{dip}=-\frac{\mu_{0}\vec{m}}{r^{3}}$. The presence of a stray field in the $-\vec{m}$ direction, which couples to the neighboring moment, results in an effective antiferromagnetic interaction between the islands.

Assuming each island is a macrospin *m_i_*, we can write the energy of an island as $E_{i}=K\cos^{2}\theta -\sum_{j}E_{dip,i,j}-m_{i}\mu_{0}H_{z}$, where *K* is the anisotropy constant and $E_{dip,i,j}=\int {\vec{B}}_{dip,j}\left(\vec{r}\right)\cdot d{\vec{m}}_{i}\left(\vec{r}\right)$ is the energy generated by the dipolar interaction. Here, ${\vec{B}}_{dip,j}\left(\vec{r}\right)$ is the integral of the field emanating from all magnetic moments in island *j* on the infinitesimal element $d{\vec{m}}_{i}\left(\vec{r}\right)$. We compare the expected magnitude of each energy term. The anisotropy constant $K=m_{i}H_{c}/2\underset{∼}{>}10$ eV^[^ ^29^ ^]^ is estimated considering the island as a macrospin (*m_i_* =  330 eV/T) that can either point up or down as our data suggests. At *H_z_* =  0, all the arrays remain magnetized in the same direction, suggesting that the magnetic anisotropy term dominates the dipolar interaction (Figure 3a). This conclusion is reinforced by finite element calculation finding the nearest neighbors *E_dip_* ≤ 1 eV in our geometry. The anisotropy can be reduced significantly by applying an external field near *H_c_* (Figure 3b). Assuming an arbitrary precision on the applied magnetic field, the energy barrier can be made arbitrarily small, ignoring randomizing effects, such as thermal activation, and assuming that all the islands are identical. Thus, assuming that the dipolar interaction is larger than the randomizing effects at *H_c_*, we expect nearest neighbors to point in opposite directions. Further investigations could combine these effective antiferromagnetic interactions, mediated by the dipolar field with other lattices that induce geometric frustration, to study artificial spin ice in magnetic 2D materials. Here, we study out‐of‐plane magnetization in a square lattice to demonstrate the antiferromagnetic interactions.

**Figure 4:**
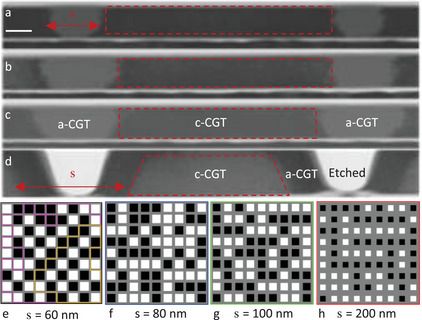
Separation‐dependent inter‐island interactions a–d) Cross‐section of scanning transmission electron microscopy images of the CrGeTe_3_ (CGT) arrays. The scale bar is 30 nm. e–h) Sequence of binary matrices computed from the SQUID‐on‐tip (SOT) *B_z_* (*x*,*y*) images for island separations of s = 60, 80, 100, and 200 nm e,f,g,h). The purple and gold lines in e delineate two fully anticorrelated sublattices. The magnetization curves *M*(*H_z_*) of the nano‐patterned arrays is presented in Figure S1 (Supporting Information). The black/white color scale represents the magnetic moments pointing down/up, and the gray represents amorphous CGT. The SOT images were acquired at the relevant coercive fields, µ_0_ *H_c_* = 68, 89, 99, and 70 mT e,f,g,h).

In order to investigate the magnetic interaction between the islands, we fabricated arrays of 9 × 9 islands. The inter‐island distance, *s*, was varied by controlling the intervening amorphous or etched area. The Ga^+^ FIB was employed to fabricate arrays with four distinct inter‐island distances (*s*  =  60,  80,  100, and 200 nm), as shown in the STEM images presented in Figure 3a–d. Three arrays (*s*  =  60,  80,  and 100 nm) were fabricated from a flake with a thickness of *d*  =  35 nm. The distance between the centers of the amorphous track in the arrays is kept constant (300 nm), resulting in an island width of *w*  =  300 − *s* nm. The array with *s*  =  200 nm was defined by etching rather than amorphization, resulting in trapezoidal islands with a width of 150 nm width and a thickness of 60 nm (**Figure** **4**d). For this array, the distance between the lines was slightly larger (350 nm). Previous work demonstrated that amorphous CGT is non‐magnetic.^[^ ^28^ ^]^ This work confirms our previous result and no difference was observed between etched and amorphized grids.

Figure 4e–h depicts the binary representation of the magnetic arrays at the coercive field, *H_c_*, where *M*( *H_z_* = *H_c_* ) ≈ 0. The emerging magnetic patterns for *s*  =  60 nm (Figure 4e), reveal a propensity for each island to orient the magnetization in a direction antiparallel to its neighbors. Conversely, as observed in Figure 4h (*s*  =  200 nm), more distant neighboring islands are more often magnetized in parallel to each other, forming larger magnetic domains. The field evolution of the arrays is presented in Movies S3–S6 (Supporting Information). In the discussion, we analyze the correlation and consider the relevant energy scales of the system.

## 3 Discussion

**Figure 5:**
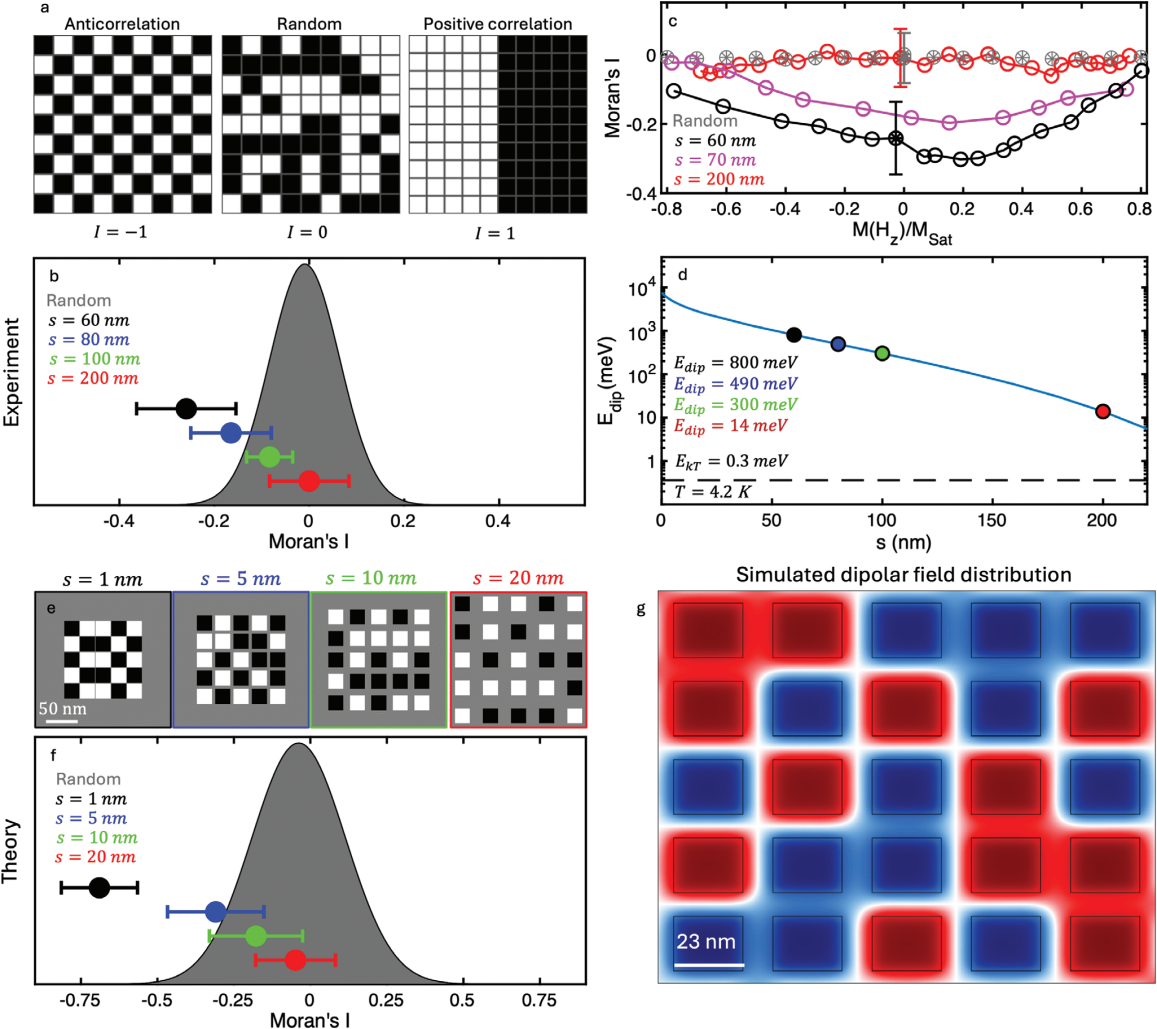
Quantitative analysis of the correlation for arrays with distinct separations, *s* a) Illustration of matrices at the coercive field (*M*(*H_z_* = *H_c_*) = 0) with different types of inter‐island correlations. A checkboard matrix that shows perfect anticorrelation with Moran's *I*  =   − 1 (left). A random matrix, which has zero spatial correlation *I* = 0 (center). A perfectly correlated matrix for which *I* = 1. b) Gray: distribution of Moran's *I* values calculated for random 9 × 9 matrices at the coercive field based on a set of 10^4^ trials. Distinct markers represent the average Moran's *I* measured for CrGeTe_3_ (CGT) arrays at *H_c_* for separations of *s* = 60 (black), 80 (blue), 100 (green), and 200 (red) nm. The markers indicate the mean values and the error bars are the range within one standard deviation. The number of repetitions varies for each sample, being 8, 8, 7, and 5, respectively. *I* = 0.00 ± 0.08,   − 0.08 ± 0.05,   − 0.16 ± 0.08,  and − 0.25 ± 0.1 for *s* = 100,  80 and 60 nm, respectively. c) Magnetization dependence of Moran's *I* value for *s* = 60 (black), *s* = 70 (magenta),  200 nm (red), and computer‐generated random matrices (grey). The magnetization is normalized to that of the fully magnetized array, *M_Sat_*. The number of repetitions for each curve is 8 (red), 1 (magenta), and 3 (black) loops. The results for *s* = 70 nm are taken from Figure 2. The error bars are representative of all data points measured for a given array. d) The dipolar energy, *E_dip_*, (light blue line) between two islands with a separation *s* obtained from finite element calculations. A constant distance of 300 nm was assumed, resulting in an island size of *w* = 300 nm − *s* and a thickness of *d* = 35 nm. The thermal energy, *E_kT_*, at *T* = 4.2 K is included for reference (dashed black line). e–g) Atomistic spin dynamics simulations for 5×5 CGT arrays with distinct separations *s*. e). Illustration of matrices at the coercive field (*M*(*H_z_* = *H_c_*) = 0) with island dimension of 23 × 23 × 3 nm^3^ and s = 1, 5, 10, and 20 nm. The black/white color scale represents the magnetic moments pointing down/up and the gray represents nonmagnetic CGT. f). Gray: distribution of Moran's *I* values calculated for random 5 × 5 matrices at the coercive field based on a set of 10^4^ trials. Distinct markers represent the average Moran's *I* values of CGT arrays at *H_c_* for separations of *s* = 1 (black), 5 (blue), 10 (green), and 20 (red) nm. The markers indicate the mean values, and the error bars are the range within one standard deviation. Each data point represents the average of ten iterations. *I* = −0.05 ± 0.1, −0.2 ± 0.2,   − 0.3 ± 0.2,  and − 0.7 ± 0.1) for *s* = 20,  10,  5 and 1 nm respectively g). Simulated spatial distribution of the *z*‐th component of the demagnetizing field, $B_{z}^{\mathrm{demag}}$, for a 5 × 5 squared array composed of rectangular grains of 23 × 23 × 3 nm^3^ for an inter‐island separation of *s* = 10 nm, which depicts a remarkable anticorrelation.

We now quantify the strength of this AFM correlation as a function of the inter‐island separation, *s*. To determine the spatial autocorrelation within the matrices depicted in Figure 4, we employ the Moran's *I* metrics.^[^ ^30^, ^31^ ^]^ *I* is calculated from the following expression:

(1) $$I=\frac{n}{\sum_{i}\sum_{j}w_{i,j}}\;\frac{\sum_{i}\sum_{j}w_{i,j}\left(m_{i}-\bar{M}\right)\left(m_{j}-\bar{M}\right)}{\sum_{i}{\left(m_{i}-\bar{M}\right)}^{2}}$$

where, *n* is the number of islands, *m_i_*,*m_j_* refer to the island magnetization in the location *i*, *j*, $\bar{M}=M\left(H_{z}\right)/M_{Sat}$ is the normalized mean value of the array magnetization, and *w*_*i*,*j*_ is the spatial weight between cells. To consider the nearest neighbor correlation, we set *w*_*i*,*j*_ =  1 between nearest neighbors, while all the other *n* ^2^ × *n* ^2^ matrix entries are set to zero. The *I* values range between −1 and 1, with a value of ‐1 indicating a perfect negative correlation and a dominant AFM interaction (**Figure** **5**a, left panel). *I*  =  0 indicates the absence of any correlation, as expected for a randomly distributed matrix (Figure 5a, central panel). A value of 1 is obtained in the case of perfect positive correlation (ferromagnetic interaction), which would result in two magnetic domains pointing in opposite directions (Figure 5a, right panel). We note that this metric is also useful away from the coercive field but is not defined for a fully magnetized array (*M* (*H_z_*) = *M_Sat_* ) since the denominator, $\sum_{i}{\left(m_{i}-\bar{M}\right)}^{2}$, vanishes when $m_{i}=\bar{M}\;\forall \;i.$

Figure 5b presents a distribution of Moran's *I* values derived from computer‐generated 9 × 9 random matrices at *H_c_* (*M*( *H_z_* = *H_c_* )  =  0) and based on a set of 10^4^ trials (gray distribution). This distribution is centered around *I*  =  0, highlighting zero spatial correlation in the absence of dipolar interactions. Each marker plotted in Figure 5b represents the experimentally determined mean *I* values at *H_c_*, obtained from distinct field sweeps. The error bars represent the obtained standard deviation. For the largest separation (*s*  =  200 nm), we obtain *I*  =  0.00 ± 0.08, which indicates negligible interaction (Figure 5b, red marker). Conversely, significant anticorrelations between the islands are observed for *s* < 100 nm (*I*  =   − 0.08 ± 0.05,   − 0.16 ± 0.08,  and − 0.25 ± 0.1, for *s*  =  100,  80, and 60 nm, respectively).

Figure 5c presents Moran's *I* values as a function of the magnetization, *M*(*H_z_*) of the array. The gray points indicate values derived from random matrices, based on 10^3^ trials for each *M*(*H_z_*) value. We restrict ourselves to |*M*(*H_z_*)|/*M_Sat_* < 0.8 because the metric diverges at |*M*(*H_z_*)|/*M_Sat_* =  1. For *s*  =  200 nm, the interaction appears weak (*I* ≈ 0) for all *M*(*H_z_*) values, which resembles the situation in the random case. For *s*  =  60 nm, at (|*M*(*H_z_*)|/*M_Sat_* ≈ 0.8), we obtain *I* ≈ 0, which suggests little anticorrelation for a relatively large magnetization state. In contrast, low magnetization, |*M*(*H_z_*)|/*M_Sat_* < 0.8, is associated with clearly negative *I* values, indicating that dipolar interactions induce AFM correlations that vanish at stronger applied magnetic fields. We have also computed and plotted Moran's *I* for an array of 23 × 23 islands with a separation of *s*  =  70 nm, as shown in Figure 2 (Figure 5c, magenta). As anticipated, the array exhibits a substantial interaction, albeit weaker than that observed for separation of *s*  =  60 nm.

The anticorrelations revealed by our results are significant but not dominant since *I* < 0.3. This can be attributed to randomizing effects such as finite‐temperature fluctuations or inhomogeneous island properties. These fluctuations could explain the finite range of fields at which the magnetization reverses. In the absence of inhomogeneity and thermal fluctuations, all islands should reverse their magnetization at the same field. In contrast, we observe consistently a transition over a field range of 70 mT (Figure 2g; Figure S1, Supporting Information). Another source of disorder is the two‐fold degeneracy of the AFM lattice (Figure 4e). In previous works, the application of demagnetization^[^ ^32^, ^33^ ^]^ or thermal^[^ ^34^ ^]^ protocols was used to relax the system into the ground state. This is visible in Figure 4e where the top right part and the bottom left part are locked in different AFM domains. More than 80% of the nanoislands are part of these two domains and most of the remaining nanoislands are located at the grid's edge where there is a reduced number of neighbors.

The effect of thermal fluctuations is also noticeable when imaging the grid at the same field for an extended period of time. The effect of thermal activation is visible in Movie S7 (Supporting Information), where we measure at a fixed field every 15 minutes during a 6 h period. We notice that during that time, the magnetization evolved from *M*/ *M_tot_* =   − 0.4 to − 0.1 (Figure S2, Supporting Information). Thermal relaxation also allows the grid to approach a more anticorrelated state as revealed by the value of Moran's *I*, which evolves from − 0.10 to − 0.17. The thermal activation at 4.2 K suggests that the thermal annealing procedure would be beneficial to enhance anticorrelations. It demonstrates that thermal activation is a relevant energy scale at 4.2 K near the coercive field. However, at zero applied field the anisotropy barrier is too large (10 eV), and no thermal activation was observed.

To determine the strength of interaction, we used the finite element method to calculate the dipolar energy (see Experimental Section), *E_dip_*, between two islands with separation *s* (Figure 5d). The results reveal *E_dip_* grows by two orders of magnitude between *s*  =  200 nm and *s*  =  60 nm. This calculation explains why the Moran's *I* grows significantly with decreasing *s*. However, it cannot explain the observed variation in *H_c_* since $K\underset{∼}{>}10\;\mathrm{eV}$ and *E_dip_* ≤ 0.8 eV (Table S1). The measured variations in *H_c_* depend on the island dimensions and not on the spacing *s* as discussed elsewhere.^[^ ^35^ ^]^ Improving the uniformity of the island properties and enhancing *E_dip_* is achievable by using a higher resolution amorphization tool such as He FIB.^[^ ^36^ ^]^ This would also allow us to decrease the separation to *s*  =  10 nm, which would increase the dipolar energy by nearly an order of magnitude.

To explore which kind of response would be physically expected at smaller spatial scales than those achievable experimentally, we have generated squared arrays of CGT‐based islands of dimension 23 × 23 × 3 nm^3^ in the framework of atomistic spin dynamics simulations^[^ ^37^, ^38^, ^39^, ^40^ ^]^ (see Experimental Section). The selected dimensionality of the modeled flattened islands maintains the same aspect ratio as in the experimental case but scaled down by an overall factor of ≈ 10. We initially left the system to equilibrate by 5 × 10^5^ Monte Carlo steps at a constant temperature of *T*  =  90 K, well above the simulated Curie temperature (*T*_C_ =  61.1 K). Afterward, a zero‐field cooling process was carried out for 10^6^ steps, where the temperature was linearly reduced from an initial state (*T*  =  90 K) to the targeted final value (*T*  =  0 K). Once this stage was reached, the system was allowed to evolve for 2.5 × 10^5^ extra steps with the aim of ensuring good convergence in the final state. Throughout the entire process, the demagnetizing contribution has been characterized through the inter‐intra dipole tensor approach aiming for computational efficiency.^[^ ^41^ ^]^

Within this computational framework, we preliminarily explore the emergence of anticorrelation in arrays with different numbers of CGT‐based magnetic islands separated by a distance (*s*). Interestingly, we found that for grids of a 3 × 3 array, or smaller, no sizable dipolar‐induced AFM interactions between the islands were present. The systems stabilized in a random orientation despite of the initial spin states and magnitudes of *s*. This is attributed to the fact that in a small array, a large proportion of the island is located at the edge of the array and thus has fewer neighbors. A similar effect was observed experimentally at the edge of larger array as discussed above (Figure 5e). We observe that anticorrelation effects start to be present at systems composed by squared 5 × 5 arrays with island dimension of 23 × 23 × 3 nm^3^. In this scenario, an array with *s*  =  1,  5,  10, and 20 nm (Figure 5e) clearly displays an increasing AFM coupling between the islands as *s* decreases. The Moran's *I* index computed for each configuration (Figure 5f) followed the same trend observed in the experiments (Figure 5b) at a much smaller spatial scale. We note that the anticorrelation is significantly larger than in experiments due to the small spacing. This indicates that similar phenomena are present in both situations. Furthermore, the spatial distribution of the normalized out‐of‐plane *z*‐th demagnetization field component ($B_{z}^{\mathrm{demag}}$) for *s*  =  10 nm (Figure 5g) remarkably demonstrated that dipolar interactions are the main driving‐force for the anticorrelation across the magnetic grid. Neighboring islands with a parallel magnetization (e.g., blue‐blue or red‐red) tend to be ferromagnetically coupled, whereas anti‐parallel configurations (e.g., blue‐red) shows that the demagnetizing field canceled out in the middle region between the islands. In the limit of a full anticorrelation (Moran's *I* ≈ −1), the magnetic grid would develop zero stray fields ($B_{z}^{\mathrm{demag}}=0$) between the islands with resemblance of a chess‐board pattern (Figure S3, Supporting Information). Simulations at higher temperature resulted in a *I* closer to zero highlighting the importance of minimizing thermal fluctuations (Figure S4, Supporting Information).

In conclusion, we have demonstrated that by nanopatterning a 2D vdW magnet in a square grid we can finely adjust interactions to create states not accessible in isolated pristine flakes, which exhibit zero net magnetization at zero field. Such lab‐made structures provide an exciting playground to investigate a wide class of fundamental correlated phenomena in CGT and in other 2D vdW magnets. We note that the dipolar coupling generates a rich type of interaction. For example, a material with in‐plane anisotropy would result in a ferromagnetic interaction along the magnetic moment and an antiferromagnetic interaction along the perpendicular direction. Further investigations could combine the effective antiferromagnetic interactions, mediated by the dipolar field with other lattices that induce geometric frustration to study artificial spin ice in magnetic 2D materials. These phenomena are not beyond reach thanks to lithography techniques, and the flexibility in sample fabrication using vdW material technology. As opposed to vertical approaches, commonly used through stacking layers on top of each other following different protocols (e.g., moiré stacking, multiple assemblies, intercalation), the lateral engineering of magnetic layers provides an intriguing platform to generate disparate structures with tunable behavior. Our results open pathways to connect underlying results in spin‐ice systems, frustration physics, and criticality to the ultimate thin limit routinely achieved in 2D materials but not so in more traditional magnets.

## 4 Experimental Section

### Sample Synthesis

Single crystals of CrGeTe_3_ were grown using slight excess of Te.^[^ ^42^, ^43^ ^]^ These samples were grown from high‐purity Cr (Alfa Aesar 99.999%), Ge (GoodFellow 99.999%) and Te (GoodFellow 99.999%). Cr, Ge and Te were introduced and sealed in quartz ampoules. Then, the ampoules were heated from room temperature to 930 °C in 12 h, cooled down to 715 °C in 54 h, and finally cooled down to 500 °C in 54 h; here the samples remain during 99 h. The crystals down to ambient temperature by immersion in cold water were quenched. Layered crystals of 2 mm × 2 mm × 0.5 mm were obtained.

### Sample Fabrication

CGT samples were fabricated using the dry transfer technique, which was carried out in a glovebox with an argon atmosphere. The CGT flakes were cleaved using the scotch tape method and exfoliated on commercially available Gelfilm from Gelpack. The CGT flakes were transferred onto a SiO_2_ substrate. The flakes were exfoliated from the crystals in areas without any Te flux. This was achieved by optically checking that the sample area was free of any inclusions and had large and flat surfaces. The various island shapes were etched or amorphized using a 30 keV Ga^+^ focused ion beam (FIB).^[^ ^28^ ^]^

### Scanning SQUID‐On‐Tip Microscopy

The SOT was fabricated using self‐aligned three‐step thermal deposition of Pb at cryogenic temperatures, as described previously.^[^ ^26^ ^]^ The SQUID‐on‐tip resolution is determined primarily by the largest of the following parameters: the SQUID loop diameter and the tip‐to‐sample distance. In this work, the island size was about 200 nm, therefore a tip diameter ranging from 145 to 175 nm and the distance from the sample was ranging from 50 to 200 nm was used. All measurements were performed at 4.2 K in a helium atmosphere at 1 to 10 mbar. The sample was exposed to air about one hour each time it was loaded in the microscope or to change tip. Samples are typically exposed a few hours without any noticeable change in their magnetic properties.

### Sample Characterization

High‐resolution scanning electron microscope (SEM) cross‐section lamellas were prepared and imaged by Helios Nanolab 460F1 Lite focused ion beam (FIB) – Thermo Fisher Scientific. The site‐specific thin lamella was extracted from the CGT patterns using FIB lift‐out techniques.^[^ ^44^ ^]^ STEM and Energy‐Dispersive X‐ray Spectroscopy (EDS) analyses were conducted using an Aberration Prob‐Corrected S/TEM Themis Z G3 (Thermo Fisher Scientific) operated at 300 KV and equipped with a high‐angle annular dark field detector (Fischione Instruments) and a Super‐X EDS detection system (Thermo Fisher Scientific). See Note S1 and Figure S5 (Supporting Information) for more details.

### Atomistic Spin Dynamics Methods

The modeling of the initial rhombohedral CGT pristine structure has been reduced to the characterization of the spatial hexagonal arrangement of the Cr‐based atoms in the magnetic unit cell, whose 3D dimensions are given by *a*  =  6.91 Å, *b*  =  11.97 Å, and *c*  =  21.82 Å. In order to successfully capture the physical response of the system, the configurational spin Hamiltonian, H, includes:

(2) $$H=-\frac{1}{2}\sum_{i\neq j}m_{i}I_{ij}m_{j}-K\sum_{i}{\left(m_{i}\cdot \hat{z}\right)}^{2}-\mu_{s}\sum_{i}\left(m_{i}\cdot B_{i}^{\mathrm{demag}}\right)$$

where **m**_*i*_ and **m**_*j*_ represent the magnetization vectors at the *i*‐ and *j*‐th atomic positions, normalized by the atomic magnetic moment, µ_*s*_. The symmetric exchange parameter, $I_{ij}$, accounts for the bilinear intra‐ ($I_{1}=7.52$ meV, $I_{2}=-0.16$ meV, and $I_{3}=0.32$ meV), and interlayer ($I_{z1}=-0.10$ meV, $I_{z2}=0.24$ meV, and $I_{z3}=0.76$ meV) interactions up to the third nearest neighbors were extracted from previous simulations.^[^ ^45^ ^]^ The preferential direction for the magnetization in this material is along the *z*‐th axis with a uniaxial single‐ion anisotropy *K*  =  0.05 meV.^[^ ^25^ ^]^ The third term on Equation (2) defines the magnetic dipolar contribution, an expression which is dominated by the demagnetizing field acting on the *i*‐th magnetic atom, $B_{i}^{\mathrm{demag}}$. In this approach the calculation of the dipole interaction acting in the *i*‐th magnetic atom is computed atomistically within a selected cut‐off radius (*r*_c_) centered on the reference atom, while to find the influence of atoms outside this range the macrocell formalism is followed. The micromagnetic cuboid cells for the long‐range scenario have a fixed size, calculating for each of them their associated total magnetic moment through which they will act as effective dipoles for distant magnetic atoms. Thus, the demagnetizing field, $B_{i}^{\mathrm{demag}}$, included in Equation (2), will be calculated based on the distance of each magnetic atom to the *i*‐th reference one, |**r**_*ij*_|, as follows:

(3) $$B_{i}^{\mathrm{demag}}=\left\{\begin{array}{c} \frac{\mu_{0}}{4\pi }\sum_{i\neq j}\frac{3\left(m_{i}\cdot {\hat{r}}_{ij}\right)\left(m_{j}\cdot {\hat{r}}_{ij}\right)-\left(m_{i}\cdot m_{j}\right)}{{\left|r_{ij}\right|}^{3}},\;\mathrm{if}\;\left|r_{ij}\right|\leq r_{c},\\ \frac{\mu_{0}}{4\pi }\sum_{i\neq p}\frac{3\left(m_{i}\cdot {\hat{r}}_{ip}\right)\left(m_{p}^{\mathrm{mc}}\cdot {\hat{r}}_{ip}\right)-\left(m_{i}\cdot m_{p}^{\mathrm{mc}}\right)}{{\left|r_{ip}\right|}^{3}},\;\mathrm{if}\;\left|r_{ij}\right|>r_{c}, \end{array}\right.$$

where µ_0_ represents the vacuum magnetic permeability, $m_{p}^{\mathrm{mc}}$ the magnetic moment of the *p*‐th macrocell, and ${\hat{r}}_{ip}$ the unit vector linking the *i*‐th magnetic atom and the center of mass of the *p*‐th macrocell.^[^ ^37^, ^45^ ^]^ In the simulations, a macrocell size (1 × 1 × 1 nm^3^) and cut‐off radius were operated, which governs the transition from the atomistic to the macrocell dipolar characterization for atoms beyond two macrocells from the reference *i*‐th magnetic atom.

### Finite Element Calculation

The energy resulting from the dipolar interaction was calculated by the finite element method. The sample was assumed to be perfectly confined in the *xy* plane. Two islands of width *w* separated by *s* were considered to calculate the nearest neighbors interaction. Each island into *N* ^2^ elements of $\frac{w}{N}\times \frac{w}{N}$ was divided. For each element *i* of one island, the field generated on that element by all the other elements of the island ($\vec{B_{i}}=\frac{1}{4\pi }\;\sum_{i=1}^{N^{2}}\frac{-\vec{m_{j}}}{{|r_{ij}|}^{3}}$) was calculated. The interaction energy is obtained as follow $E_{i}=-\vec{m_{i}}\;\cdot \vec{B_{i}}$. The total energy is obtained by summing the energy calculated for each element $E_{island}=\sum_{i=1}^{N^{2}}E_{i}$.

## Conflict of Interest

The authors declare no conflict of interest.

## Author Contributions

Y.A., and A.N. conceived the experiment. E.H. and H.S. synthesized the CGT crystals. A.N., Y.Z., and N.F. carried out the scanning SOT measurements. Y.A., M.K., H. Steinberg, and A.N. fabricated the CGT devices. A.N. characterized the CGT devices. A.N., M.M., Y.K.K., T.L., and M.R.A. analyzed the data. Y.A., and A.N. constructed the scanning SOT microscope. M.E.H. developed the SOT readout system. R.R.‐E., H. Solanki, E.J.G.S., and S.G. provided theoretical and simulation support. A.N., E.J.G.S., O.M., and Y.A. wrote the paper with contributions from all authors.

## Supporting information



Supporting Information

Supplemental Movie 1

Supplemental Movie 2

Supplemental Movie 3

Supplemental Movie 4

Supplemental Movie 5

Supplemental Movie 6

Supplemental Movie 7

## Data Availability

The data that support the findings of this study are available from the corresponding author upon reasonable request.
