# Supplementary material for: Field‐Induced Antiferromagnetic Correlations in a Nanopatterned Van der Waals Ferromagnet: A Potential Artificial Spin Ice
Source: Adv Sci (Weinh). 2024 Dec 8;12(5):2409240. doi: 10.1002/advs.202409240 (PMC11791941; doi:10.1002/advs.202409240)
Supplement: Supplementary file 1 — Supporting Information [file ADVS-12-2409240-s007.docx]

**Supplementary Information**

***Field-induced antiferromagnetic correlations in a nanopatterned van der Waals ferromagnet: a potential artificial spin ice***

*Avia Noah*^1,2,3^, Nofar Fridman^1,2^*, *Yishay Zur^1,2^, Maya Markman**^1^, Yotam Katz King^1,2^, Maya Klang^1^, Ricardo Rama-Eiroa^4,5^, Harshvardhan Solanki^5^, Michael L. Reichenberg Ashby^1,6^, Tamar Levin^1^,* *Edwin Herrera^7^, Martin E. Huber^8^, Snir Gazit^1^, Elton J. G. Santos*^4,5,9^, Hermann Suderow^7^, Hadar Steinberg^1,2^, Oded Millo^1,2^, and Yonathan Anahory*^1,2^*

^1^The Racah Institute of Physics, The Hebrew University, Jerusalem, 9190401, Israel

^2^Center for Nanoscience and Nanotechnology, The Hebrew University, Jerusalem, 91904, Israel

^3^Faculty of Engineering, Ruppin Academic Center, Emek-Hefer, 40250 Monash, Israel

^4^Donostia International Physics Center (DIPC), 20018 Donostia-San Sebastián, Basque Country, Spain

^5^Institute for Condensed Matter Physics and Complex Systems, School of Physics and Astronomy, University of Edinburgh, Edinburgh, EH93FD, United Kingdom

^6^Imperial College London, Blackett Laboratory, London, SW7 2AZ, United Kingdom

^7^Laboratorio de Bajas Temperaturas, Unidad Asociada UAM/CSIC, Departamento de Física de la Materia Condensada, Instituto Nicolás Cabrera and Condensed Matter Physics Center (IFIMAC), Universidad Autónoma de Madrid, E-28049 Madrid, Spain

^8^Departments of Physics and Electrical Engineering, University of Colorado Denver, Denver, CO 80217, USA

^9^Higgs Centre for Theoretical Physics, University of Edinburgh, Edinburgh EH93FD, United Kingdom

Email: [avia.noah@mail.huji.ac.il](mailto:avia.noah@mail.huji.ac.il), [esantos@ed.ac.uk](mailto:esantos@ed.ac.uk), [yonathan.anahory@mail.huji.ac.il](mailto:yonathan.anahory@mail.huji.ac.il)


$$s=100$$

$$s=80$$

$$s=200$$

$s=60$ *nm*

**Figure S1 - Field evolution of island arrays in CrGeTe_3_.** Hysteresis curves drawn from $B_{z}(x,y)$ measured on arrays with separation ranging between $60$ and $200$ nm. The array’s coercive field $H_{c}^{a}$ is marked with black dots. The hysteresis curves were measured by ramping the field in one direction and were symmetrized to obtain the second branch of the ${M\left( H_{z} \right)}/{M_{tot}}$ curve. Curves were shifted vertically for clarity.

**Figure S2 - Thermal activation of CrGeTe_3_ array.** (**a**) Time evolution of the array magnetization at $\mu_{0}H_{z}=95$ mT. (**b**) Moran’s I over time at $\mu_{0}H_{z}=95$ mT. The data corresponds to the array shown in Figure 2, consisting of 30x30 islands with a separation of 70 nm. Measurements were taken by continuously scanning the same area, with an imaging speed of 12 minutes per image and a 5-minute pause between images after the first four images (total of 22 images).

**Figure S3 -** Simulated spatial distribution of the out-of-plane $z$-th component of the demagnetizing field, $B_{z}^{\mathrm{demag}}$, for a $5\times5$ squared array of $23\times23\times3$ $\mathrm{nm}^{3}$ rectangular islands for different inter-grain separations $s=1$, $5$, $10$, and $20$ nm after a zero-field cooling process at a final temperature of $T=0$ K. The selected seeds included in the plots correspond to those closest to the mean Moran's $I$ value for each inter-island distance $s$ included in Figure 4f. We have employed a bicubic interpolation between the islands to obtain $B_{z}^{\mathrm{demag}}$.

Bilayer $T_{c}$

$$s=1 nm$$

$$s=5 nm$$

$$s=10 nm$$

$$s=20 nm$$

**Figure S4 - Atomistic spin dynamics simulations of Moran’s I Temperature dependent.** Distinct markers represent the average Moran’s $I$ values of $5\times5$ CGT arrays at $H_{c}$ for separations of $s=1$(black), $5$(blue), $10$(green), and $20$(red) nm. The markers indicate the mean values, and the error bars the range within one standard deviation. The island dimension is $23\times23\times3$ nm^3^. Each data point represents the average of 10 iterations.


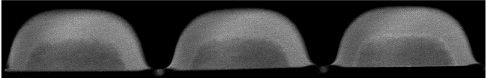


Horizontal EDS

Vertical EDS

O

Cr

Ga

b

c

a

Etched

c-CGT

a-CGT

a-CGT

c-CGT

50 nm

**Figure S5 - Scanning transmission electron microscopy and Energy-Dispersive X-ray Spectroscopy analyses of CrGeTe_3_ island.** (**a**) High-angle annular dark field (HAADF) image of the array with a separation of 200 nm. The area inside the dark red dotted line trapezoid is the region where magnetic crystalline CrGeTe3 is found. Above the crystalline region, an amorphous and non-magnetic region is observed. (**b-c**) Energy-Dispersive X-ray Spectroscopy (EDS), showing the relative amount of Cr, O, and Ga in the island cross-sections.

| **Array #** | **Island Separation**  $\boldsymbol{s}$  **(nm)** | **Effective size**  $\boldsymbol{w\times w\times d}$  **(nm^3^)** | **Volume**  $\boldsymbol{V}$  **(**$\mathbf{1}\boldsymbol{0}^{\boldsymbol{6}}$ **nm^3^)** | **Island Magnetization**  $\mathbf{m}_{\mathbf{i}}\mathbf{=}\frac{\mathbf{3}\boldsymbol{\mu}_{\mathbf{b}}\mathbf{V}}{\mathbf{V}_{\mathbf{cell}}}$  $\mathbf{(}$**eV T^-1^**$\mathbf{)}$ | **Island Magnetization**  $\mathbf{m}_{\mathbf{i}}\mathbf{=}\frac{\mathbf{3}\boldsymbol{\mu}_{\mathbf{b}}\mathbf{V}}{\mathbf{V}_{\mathbf{cell}}}$  $\mathbf{(1}\boldsymbol{0}^{\boldsymbol{6}} \boldsymbol{\mu}_{\mathbf{b}}\mathbf{)}$ | **Median island Coercivity** $H_{c}$  **(mT)** | **Dipolar Energy**  $\mathbf{E}_{\mathbf{dip}}$  **(eV)** | **Median island anisotropy**  $\mathbf{K=}\frac{\mathbf{H}_{\mathbf{c}}^{\mathbf{i}}\mathbf{M}}{\mathbf{2}}$  **(eV)** |
| --- | --- | --- | --- | --- | --- | --- | --- | --- |
| 1 | $60\pm4$ | $240\boldsymbol{\times}240\boldsymbol{\times}35$ | $2.0\pm0.1$ | $420\pm30$ | $7.3\pm0.5$ | $67\pm4$ | $0.8\pm0.1$ | $14\pm2$ |
| 2 | $70\pm4$ | $230\boldsymbol{\times}230\boldsymbol{\times}30$ | $1.6\pm0.1$ | $330\pm20$ | $5.7\pm0.5$ | $95\pm4$ | $0.5\pm0.1$ | $15\pm2$ |
| 3 | $80\pm4$ | $220\boldsymbol{\times}220\boldsymbol{\times}35$ | $1.7\pm0.1$ | $360\pm30$ | $6.2\pm0.5$ | $89\pm4$ | $0.5\pm0.1$ | $15\pm$2 |
| 4 | $100\pm4$ | $200\boldsymbol{\times}200\boldsymbol{\times}35$ | $1.4\pm0.1$ | $290\pm20$ | $5.0\pm0.3$ | $99\pm4$ | $0.3\pm0.1$ | $14\pm2$ |
| 5 | $200\pm10$ | $150\boldsymbol{\times}150\boldsymbol{\times}60$ | $1.4\pm0.1$ | $280\pm20$ | $4.8\pm0.3$ | $70\pm5$ | $0.04\pm0.1$ | $10\pm$2 |

**Table S1. A summary of the islands’ parameters and results.** The uncertainty on the dimension is $\pm5$ nm for the width $w$ and $\pm$2 nm for the thickness $d$.

**Supplementary Note 1: Sample characterization:**

Scanning transmission electron microscopy images of CrGeTe_3_ islands:

Lamellas were prepared and imaged by Helios Nanolab 460F1 Lite focused ion beam (FIB) - Thermo Fisher Scientific. The site-specific thin lamella was extracted from the CGT array using FIB lift-out techniques^[1]^. Scanning transmission electron microscopy (STEM) and Energy-Dispersive X-ray Spectroscopy (EDS) analyses were conducted using an Aberration Prob-Corrected S/TEM Themis Z G3 (Thermo Fisher Scientific) operated at 300 KV and equipped with a high-angle annular dark field (HAADF) detector from Fischione Instruments and a Super-X EDS detection system (Thermo Fisher Scientific). To determine the CGT islands’ dimensions, we performed cross-section STEM on all islands of the main text. HAADF STEM images are shown in Figure 1e and Figure 3a-d.

Energy-Dispersive X-ray Spectroscopy of CrGeTe_3_ island

In Figure S5 we present the High-angle annular dark field (HAADF) image of the $150\times150\times60$ nm^3^ island. The image resolves that the crystal structure is damaged due to the FIB etching. Near the etched area, the material is amorphous (bright gray color scale) where the crystalized CGT appears darker. The images reveal the precise thickness of the flake ($d=60\pm2$ nm) and the edge cross-section $w=150\pm5$ nm. To understand the stoichiometry of the flakes we perform an energy-dispersive spectroscopy (EDS) measurement. The EDS reveals accumulation of Ga and oxidation peaks near the amorphous edge. The concentration decays abruptly over a length of tens nm. We emphasize that Ga concentration peaks appear only in the amorphous part which we found to be non-magnetic. The Ga in the crystalline area less than 2% according to our EDS measurements. Traces of Silicon were also observed in the EDS measurements which seem to originate from organic residues from the PDMS used during the exfoliation process.

**References:**

[1] M. Sezen, in *Modern Electron Microscopy in Physical and Life Sciences*, InTech, **2016**.

**Supplementary Movies:**

**
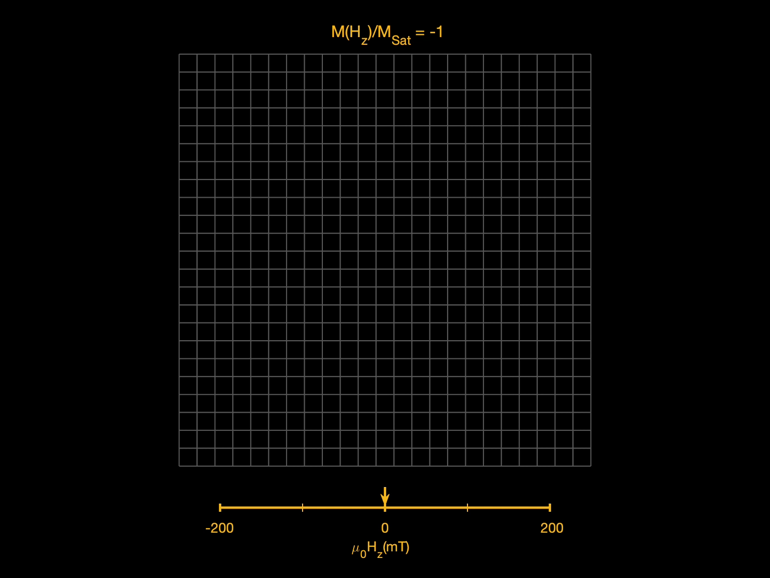
**

**Movie S1** – **Magnetic field response of the CrGeTe₃ (CGT) array magnetization.** The movie shows one magnetization loop of CGT nanoislands array, captured using SQUID-on-tip (SOT) $B_{z}(x,y)$ images at distinct values of the applied out-of-plane magnetic field $\mu_{0}H_{z}$. The images were acquired between $\mu_{0}H_{z}=\pm200$ mT. The SOT image sizes are $8\times8$ μm^2^. The black/white color scale represents the magnetic moments pointing antiparallel/parallel (down/up) to the applied field.

**
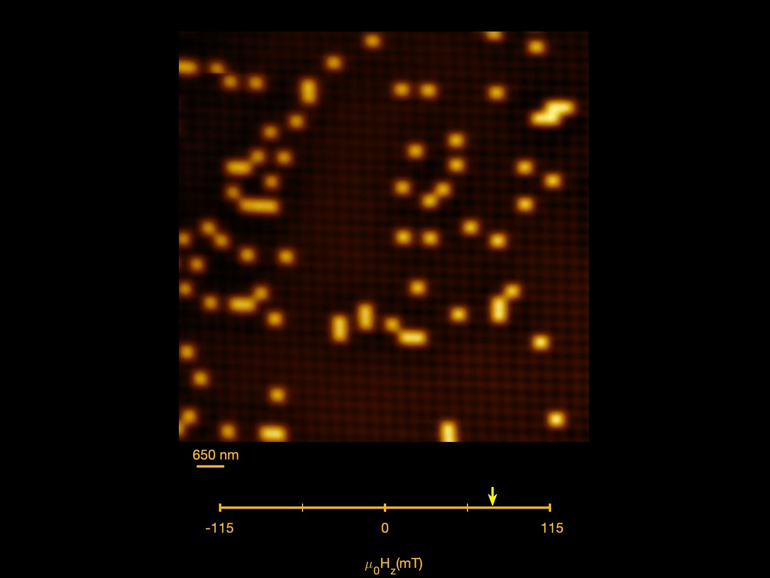
**

**Movie S2** – **Magnetic field response of the CrGeTe₃ (CGT) array magnetization.** The movie shows three magnetization loops (positive and negative) of CGT nanoislands array, captured using SQUID-on-tip (SOT) $B_{z}(x,y)$ images at distinct values of the applied out-of-plane magnetic field $\mu_{0}H_{z}$. The images were acquired between $\mu_{0}H_{z}=\pm75$ mT and $\mu_{0}H_{z}=\pm115$ mT following a field excursion of $\mu_{0}H_{z}=\mp200$ mT. The SOT image sizes are $10\times10$ μm^2^. The black/yellow color scale represents the magnetic moments pointing antiparallel/parallel (down/up) to the applied field.

**
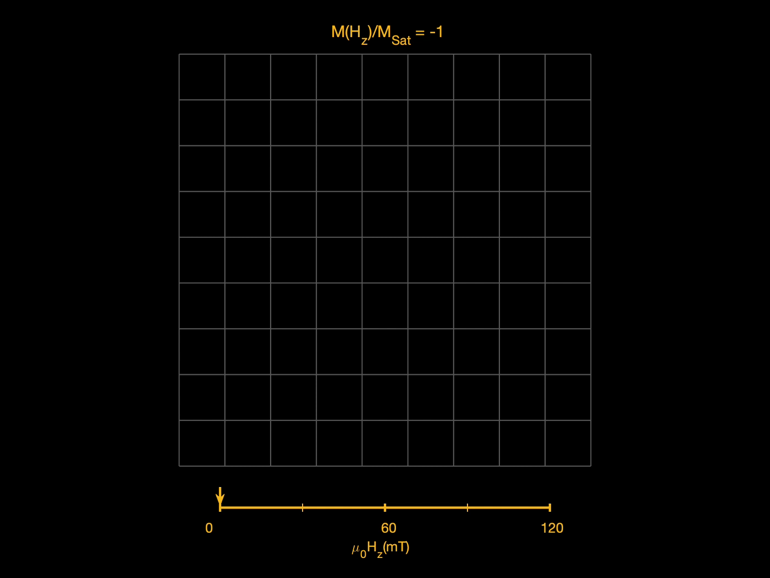
**

**Movie S3** – **Magnetic field response of the CrGeTe₃ (CGT) array with separation of 60 nm.** The movie shows the magnetic evolution of CGT nanoislands array, captured using SQUID-on-tip (SOT) $B_{z}(x,y)$ images at distinct values of the applied out-of-plane magnetic field $\mu_{0}H_{z}$. The images were acquired between $\mu_{0}H_{z}=0$ to $\mu_{0}H_{z}=120$ mT. The SOT image sizes are $5\boldsymbol{\times}5$ μm^2^. The black/white color scale represents the magnetic moments pointing antiparallel/parallel (down/up) to the applied field.

**
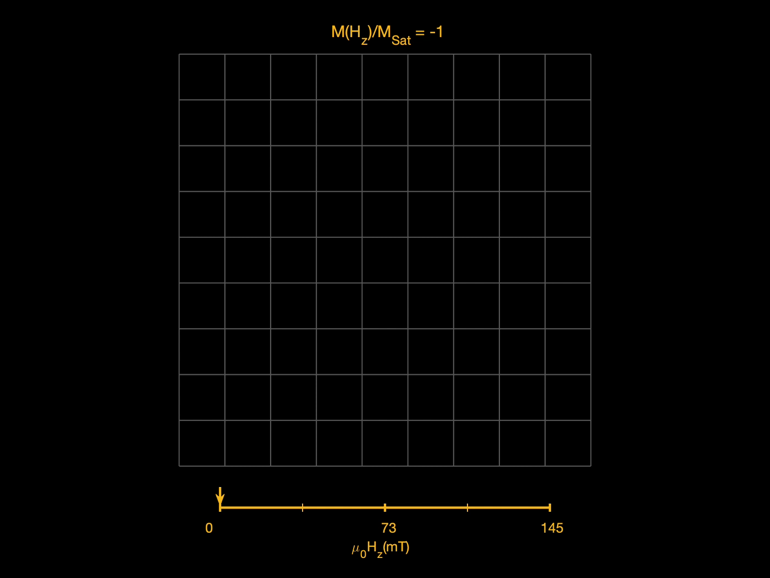
**

**Movie S4** – **Magnetic field response of the CrGeTe₃ (CGT) array with separation of 80 nm.** The movie shows the magnetic evolution of CGT nanoislands array, captured using SQUID-on-tip (SOT) $B_{z}(x,y)$ images at distinct values of the applied out-of-plane magnetic field $\mu_{0}H_{z}$. The images were acquired between $\mu_{0}H_{z}=0$ to $\mu_{0}H_{z}=145$ mT. The SOT image sizes are $5\times5$μm^2^. The black/white color scale represents the magnetic moments pointing antiparallel/parallel (down/up) to the applied field.

**
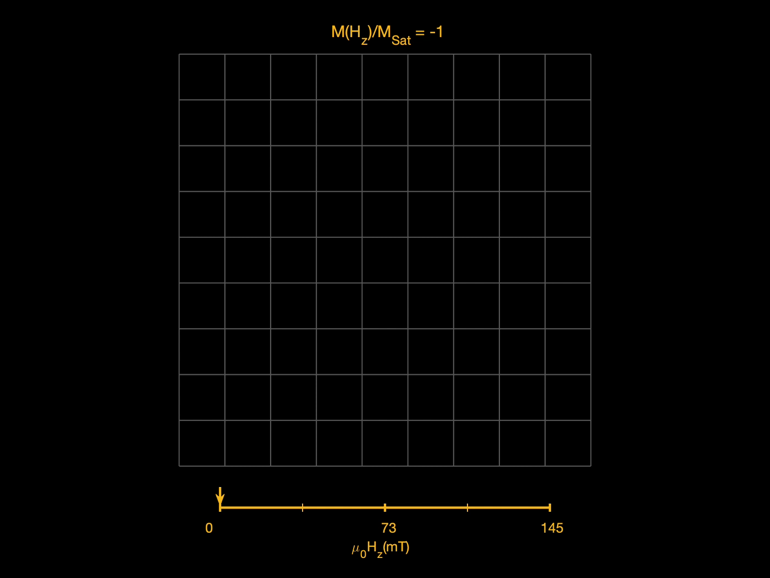
**

**Movie S5** – **Magnetic field response of the CrGeTe₃ (CGT) array with separation of 100 nm.** The movie shows the magnetic evolution of CGT nanoislands array, captured using SQUID-on-tip (SOT) $B_{z}(x,y)$ images at distinct values of the applied out-of-plane magnetic field $\mu_{0}H_{z}$. The images were acquired between $\mu_{0}H_{z}=0$ to $\mu_{0}H_{z}=145$mT. The SOT image sizes are $5\times5$ μm^2^. The black/white color scale represents the magnetic moments pointing antiparallel/parallel (down/up) to the applied field.

**
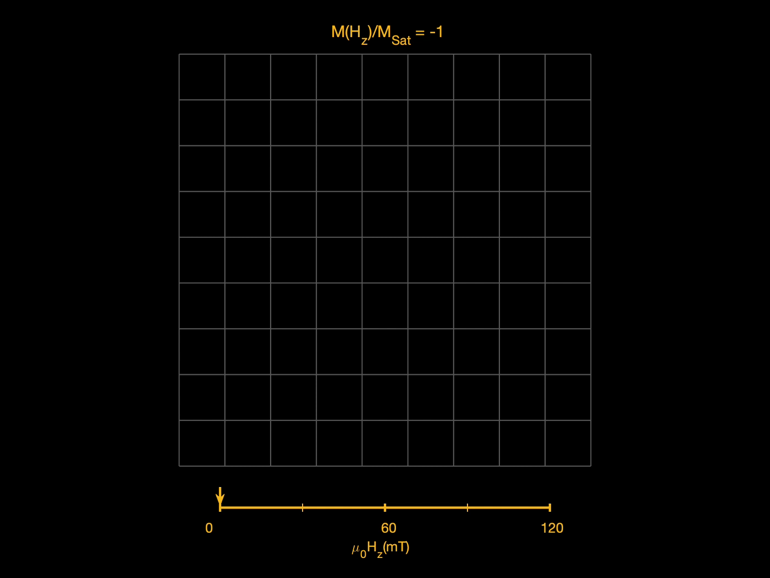
**

**Movie S6** – **Magnetic field response of the CrGeTe₃ (CGT) array with separation of 200 nm.** The movie shows the magnetic evolution of CGT nanoislands array, captured using SQUID-on-tip (SOT) $B_{z}(x,y)$ images at distinct values of the applied out-of-plane magnetic field $\mu_{0}H_{z}$. The images were acquired between $\mu_{0}H_{z}=0$ to $\mu_{0}H_{z}=120$ mT. The SOT image sizes are $4\times4$ μm^2^. The black/white color scale represents the magnetic moments pointing antiparallel/parallel (down/up) to the applied field.

**
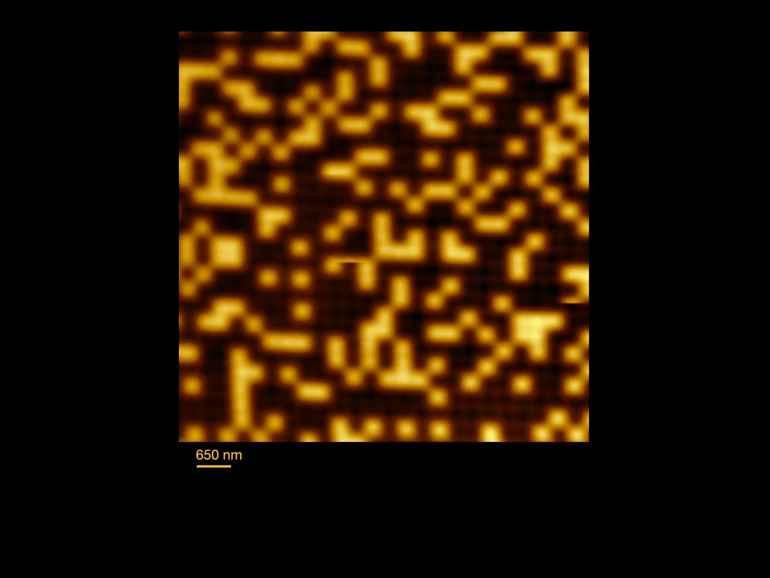
**

**Movie S7** – **Thermal activation of CrGeTe_3_ array.** Time evolution of the array magnetization at $\mu_{0}H_{z}=95$ mT. The data corresponds to the array shown in Figure 2, consisting of 30x30 islands with a separation of 70 nm. Measurements were taken by continuously scanning the same area, with an imaging speed of 12 minutes per image and a 5-minute pause between images after the first four images (total of 22 images).
